# Periodontal disease and preterm delivery: a nationwide population-based cohort study of Taiwan

**DOI:** 10.1038/s41598-022-07425-8

**Published:** 2022-02-28

**Authors:** Ya-Ling Lee, Hsiao-Yun Hu, Sin-Yi Chou, Chen-Li Lin, Feng-Shiang Cheng, Chia-Yi Yu, Dachen Chu

**Affiliations:** 1 Department of Dentistry, Taipei City Hospital, No.145, Zhengzhou Rd., Datong Dist., Taipei City, 10341 Taiwan, ROC; 2 Department of Dentistry, School of Dentistry, National Yang-Ming Chiao Tung University, Taipei, Taiwan, ROC; 3 University of Taipei, Taipei, Taiwan, ROC; 4 Department of Education and Research, Taipei City Hospital, Taipei, Taiwan, ROC; 5 Institute of Public Health, National Yang-Ming Chiao Tung University, Taipei, Taiwan, ROC; 6 Department of Obstetrics and Gynecology, Taipei City Hospital, Taipei, Taiwan, ROC; 7 Institute of Hospital and Health Care Administration, National Yang-Ming Chiao Tung University, Taipei, Taiwan, ROC; 8 Department of Neurosurgery, Taipei City Hospital, Taipei, Taiwan, ROC

**Keywords:** Periodontics, Preterm birth

## Abstract

Preterm delivery of low-birth weight infants is considered a leading cause of morbidity and mortality among neonates. Various studies have reported a positive correlation between periodontal disease (PD) and premature birth (PB) and yet no population-based study has assessed the impact of PD severity and treatments on premature birth. This cohort study used Taiwan’s national medical records (1999–2012, included 1,757,774 pregnant women) to investigate the association between PD severity and PB. Women with PD during the 2-year period prior for giving birth were more likely to have PB (11.38%) than those without PD (10.56%; p < 0.001). After variables adjustment, the advanced PD group had OR of 1.09 (95% CI 1.07–1.11) for PB, the mild PD group had OR of 1.05 (95% CI 1.04–1.06), while no-PD group had OR of 1. Increased PD severity was related to higher risk of PB. When stratified by age, the highest ORs for PB were those aged from 31 to 35 years in both mild PD group (OR = 1.09, 95% CI 1.07–1.11) and advanced PD group (OR = 1.13, 95% CI 1.09–1.17). Improving periodontal health before or during pregnancy may prevent or reduce the occurrence of adverse pregnancy outcomes and therefore maternal and perinatal morbidity and mortality.

## Introduction

A preterm delivery or premature birth (PB) entails an infant with a gestational age (GA) of less than 37 weeks at delivery^1^. According to a systematic analysis corporate with the World Health Organization (WHO) an estimation of 14.9 billion of infants were born preterm, which consist of 11.1% of newborn babies worldwide^2–5^ and approximately 10% of births in Taiwan were premature in 2019^6^. Despite advances in reproductive and neonatal medicine, the preterm birth rate has not declined, and no treatments are effective in prolonging pregnancy to prevent preterm delivery. Preterm delivery of low-birthweight (LBW) infants is considered a leading cause of morbidity and mortality among neonates, and it is a major global public health concern.

The short-term health outcomes for PB babies including respiratory distress, chronic lung diseases, apnea, feeding difficulties, necrotizing enterocolitis, gastroesophageal reflux, immature immune system and cardiovascular disorder, as a consequence, PB babies often need neonatal intensive care unit (NICU) for close monitor of health signs^7^. An observational study in Denmark suggested that premature infants under 32 weeks receiving standard care in the NICU cost average of 14,300 euros^8^. As in Canada, a cost average of $30,572 for infants with GA of 29–32 weeks; and $100,440 for those with GA of < 29 weeks. In the United stated, hospital costs averaged $202,700 for babies at 25 weeks GA, substantially decreased to $1100 for 38 weeks GA infants^9^. Studies also showed that mothers caring for preterm infants may experience heightened levels of stress, anxiety, and depression in both during and beyond NICU stay^10^. Mother who give birth to PB babies need an average of 3 years to withdraw from work compared to mother who give birth to full GA infants in order to take a close eye on their infants^11^. As for long term complication, PB babies who survived, there was a higher risk of cerebral paralysis, neurodevelopmental disorders, mental retardation, epilepsy, respiratory system diseases, pathologic heart conditions, also disorders of psychological development, behavior, and emotion, as well as disabilities that affecting work and social capacities while adulthood^12,13^. Expenses for PB babies are high in regards of neonatal intensive care and continuous medical care and education fees. The cost of medical care for premature infants impose an economic burden on families and health care systems, additionally to the emotional impact on parents^14^.

Multiple factors have been reported to be associated with PB, such as high or low maternal age, a relatively low family socioeconomic stratum, poor nutritional status, smoking, drug use, hypertension, diabetes, genitourinary tract infection, cervical incompetence, multiple pregnancies, and stress^15^. Infection and inflammation have also been proposed as causes of preterm rupture of membranes and preterm delivery^16^. Both generalized and localized infections of the genitourinary system might affect gestational length and lead to premature delivery^17,18^.

Periodontal disease (PD), caused by Gram-negative anaerobic bacteria, is a highly prevalent disease characterized by chronic inflammation. PD affects 10% to 60% of the global population and destroys periodontal supporting tissue, resulting in tooth mobility and even tooth loss^2,3,19^. Causal relationships between PD and a variety of systemic diseases have been studied. PD increases the risk of ischemic stroke, acute myocardial infarction, esophageal cancer, rheumatoid arthritis^20^, and dementia^21,22^. The relationship between PD and adverse pregnancy outcomes has also been discussed^23–25^.

Higher levels of progesterone and estrogen are produced during pregnancy, which increases periodontal vascular permeability and the amount of crevicular fluid and causes gingival edema, swelling, and inflammation^26^. A tendency to develop gingivitis during pregnancy increases the prevalence of PD. Proinflammatory mediators such as prostaglandins, cytokines, and interleukin I, induced by oral flora, also cause advanced PD^27^. Although studies have reported a positive correlation between PD and adverse pregnancy outcomes—for example, preterm delivery^28–30^, others have reported otherwise^23,31^.

To our knowledge, no population-based study has assessed the impact of PD severity and PD treatments on premature delivery. This cohort study used national medical records retrieved from Taiwan’s National Health Insurance Research Database (NHIRD) to investigate the association between PD severity and preterm delivery.

## Methods

### Data source

The investigators conducted a retrospective nationwide cohort study by analyzing data in Taiwan’s NHIRD from 1999 to 2012. The NHIRD contains more than 99% of the medical claims data of 23 million residents of Taiwan. The research data were obtained from the NHIRD program, managed by the Health and Welfare Data Science Center, Taiwan to provide useful epidemiological information for basic and clinical research in Taiwan. This database contains demographic information and claims data, including diagnoses and procedure codes. Diagnoses recorded in the database are coded according to the *International Classification of Diseases, Ninth Revision, Clinical Modification (ICD-9-CM)*. All information allowing a specific patient to be identified was encrypted^32^.

### Study cohort

The investigation included 1,757,774 pregnant women who first gave birth (primiparas) during the 13-year study period. The targeted subjects were separated into no-PD and PD groups depended on being diagnosed with PD or not. Furthermore, the researchers used *ICD-9-CM* and PD treatment codes to define PD severity and assembled into mild PD and advanced PD groups. The ages of the women ranged from 20 to 45 years.

### Definition of PD

PD is caused by specific bacterial biofilm which accumulates and forms non-calcified dental plaque and calcified dental calculus around the teeth. The micro-bacterial biofilm can induce gingival inflammation, alveolar bone destruction and even tooth loss if without optimal oral hygiene maintenance or PD treatments. The PD subjects diagnosed with gingivitis, acute or chronic periodontitis were coded as 523.0–523.5 in the *ICD-9-CM*. These codes represent various PD stages and symptoms, which can appear simultaneously in multiple areas around oral cavity. All subjects who received any PD treatment were identified with the *ICD-9-CM* code for PD in the NHI program. The subjects who suffered from advanced PD with teeth removal required, the diagnosis would be PD rather than other dental diseases, for example, tooth caries or trauma. However, *ICD-9-CM* codes do not imply any particular level of inflammation of PD. In order to assign subjects to PD groups, the researchers used the most intensive PD treatment received by the patients as the indicator for grading the severity of PD. Dentists would chart a subject’s periodontal probing depth and check full-mouth dental X-rays as baseline records before treatment. If the subjects with gingivitis or mild PD, non-invasive treatments were preformed such as local periodontal emergency treatment or dental prophylaxis. On the other hand, if the subject meets the criteria (periodontal probing depth > 5 mm or evidence of alveolar bony destruction) for moderate to advanced PD, more intensive treatments such as subgingival curettage, root planning, periodontal flap surgery or tooth extraction will be necessary^33,34^. Thus, the different periodontal treatment codes could imply the severity of PD (dental prophylaxis < PD intensive treatment and tooth extraction due to PD).

**Figure 1:**
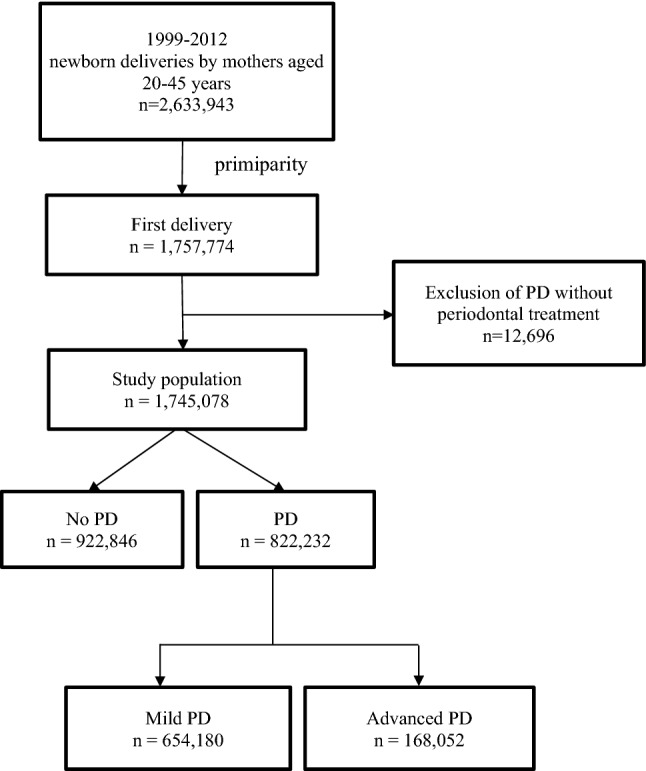
Flowchart of study population selection.

The subjects were separated into no-PD (n = 922,846) and PD (n = 834,928) groups according to *ICD-9-CM* code from 523.0 to 523.5. Those with a PD diagnosis who did not receive PD treatment (n = 12,696) were excluded from the PD group due to unable to grading their PD severity from any PD treatment that they received. Those who received periodontal treatments (n = 822,232) were separated into mild and advanced PD subgroups according to the most advanced PD treatment they received within 2 years prior to the day of their first birth delivery. The mild PD group (n = 654,180) received local emergency periodontal treatment or dental prophylaxis (tooth scaling). The advanced PD group (n = 168,052) received more intensive periodontal treatment (eg, subgingival curettage, root planning, periodontal flap operation, or tooth extraction due to severe PD; Fig. 1).

### Measures of dependent variables

The subjects diagnosed as having PB were coded as *ICD-9-CM* 644, 765.0, 765.1, or 765.20 to 765.28.

### Other confounding variables

Data on outpatient visits for multifetal pregnancy (*ICD-9-CM* 651), placental abnormalities (*ICD-9-CM* 641), diabetes mellitus complicating pregnancy (*ICD-9-CM* 648.0–648.8), vaginal infection (*ICD-9-CM* 616), autoimmune diseases (*ICD-9-CM* 710 and 714), hypertension complicating pregnancy (*ICD-9-CM* 642), or psychological factors (*ICD-9-CM* 290–319) within pregnancy period before delivery were included as individual obstetric histories. Other variables included were age and monthly income. The women were separated into 4 age groups (20–25, 26–30, 31–35, and > 35 years). Monthly income comprised 4 categories according to documented monthly income: dependent, < NT$20,000, NT$20,000–$39,999, and ≥ NT$40,000 (NT$32 = approximately US$1). Homemakers and unemployed workers were classified as dependent. Additionally, people without a documented monthly income (self-employed, retired, small shop proprietor, or those with a low income) can enroll in the health insurance scheme through labor unions or associations (e.g., farmer associations) or through local government offices, and they were assigned to the low monthly income group with an insurable wage of less than NT$20,000^35^.

### Ethical approval

The study received full approval from the Taipei City Hospital Institutional Review Board (IRB no. TCHIRB-10603107-E). The institutional review board waived the requirement for written informed consent, as all patient-identifying information was encrypted. All methods in this study were performed in accordance with relevant guidelines and regulations. This study is also in accordance with the Declaration of Helsinki.

### Data analysis

Baseline characteristics of participants were compared non-premature birth and premature birth using the chi-square test for categorical variables. The predictors for PB were analyzed using multivariate logistic regression, and adjusted for all other confounding factors, including mother’s age, monthly income, individual obstetric history, and PD condition. Stratified logistic regression analysis by age group was used to estimate the effect of PD on PB. All statistical analyses were performed using the SAS (version 9.4; SAS Institute, Inc., Cary, NC) statistical software package.

## Results

### The maternal age impact to PB

Among the 1,745,078 puerperae, 191,036 had PB delivery, resulting in an overall PB rate of 10.95%. The group aged over 35 years had the highest PB rate (12.46%); that with the lowest rate (10.34%) was the 26 to 30-year-old group (p < 0.001) The PB rate (11.39%) in the 20 to 25-year-old group was higher than in the group comprising those aged 25 to 35 inclusive (Table 1).

**Table 1:** Characteristics of study population.

Variable	Nonpremature birth		Premature birth		p value^a^
	n = 1,554,042	Rate (%)	n = 191,036	Rate (%)	
**Mother age (years)**					
20 ≤ Age ≤ 25	368,046	88.61	47,315	11.39	< 0.001
25 < Age ≤ 30	629,297	89.66	72,566	10.34	
30 < Age ≤ 35	426,998	89.01	52,698	10.99	
Age > 35	129,701	87.54	18,457	12.46	
**Monthly income**					
Dependent	354,617	89.03	43,685	10.97	< 0.001
< NT$20,000	465,966	89.43	55,076	10.57	
NT$20,000–$40,000	522,330	89.23	63,016	10.77	
> NT$40,000	211,129	87.83	29,259	12.17	
**Individual obstetric history**					
Multifetal pregnancy	6,906	47.21	7,721	52.79	< 0.001
Placental abnormality	153,632	83.18	31,057	16.82	< 0.001
Diabetes mellitus complicating pregnancy	190,966	85.36	32,745	14.64	< 0.001
Vaginal infection	1,391,265	88.99	172,105	11.01	< 0.001
Autoimmune disease	482,45	85.87	7,942	14.13	< 0.001
Hypertension complicating pregnancy	30,975	71.70	12,224	28.30	< 0.001
Psychological factor(s)	306,618	87.03	45,683	12.97	< 0.001
**Periodontal disease**					
No	825,399	89.44	97,447	10.56	< 0.001
Yes	728,643	88.62	93,589	11.38	
**Periodontal disease**					
No PD	825,399	89.44	97,447	10.56	< 0.001
Mild PD	580,190	88.69	73,990	11.31	
Advanced PD	148,453	88.34	19,599	11.66	

^a^p values were obtained from Chi-squared test for categorical variables.

### The association between incomes and PB

The risk of PB in those with a monthly income of ≥ NT$40 000 was higher than in the groups with lower monthly incomes (PB rate = 12.17% vs 10.97%, 10.57%, and 10.77% in the dependent, < NT$20 000, and NT$20 000–$39 999 groups, respectively; p < 0.001).

### The impact of comorbidity to PB

Women with multifetal pregnancy had the highest PB rate (52.79%). Those with a placental abnormality (PB rate = 16.82%), gestational diabetes (PB rate = 14.64%), vaginal infection (PB rate = 11.01%), autoimmune disease (PB rate = 14.13%), hypertension complicating pregnancy (PB rate = 28.30%), or psychological disease (PB rate = 12.97%) had higher rates of PB (p < 0.001) than those without comorbidities.

### Role of periodontal disease in preterm birth

Women with PD during the 2-year period prior to giving birth were more likely to have PB (11.38%) than those without PD (10.56%; p < 0.001). PB for those with advanced PD was more likely than for those with mild PD (11.66% vs 11.31%; p < 0 0.001; Table 1).

After adjusting for age, monthly income, and individual obstetric history, the pregnant women with PD had a higher risk of PB (odds ratio [OR] = 1.06, 95% confidence interval [CI 1.05–1.07; Appendix Table A1). The ORs of PB were further compared among the no-PD, mild PD, and advanced PD groups; after adjustment for other variables, the advanced PD group had an OR of 1.09 (95% CI 1.07–1.11), the mild PD group an OR of 1.05 (95% CI 1.04–1.06), and the no-PD group an OR of 1 (Table 2).

**Table 2:** Univariate and multivariate logistic regression of factors associated with preterm birth.

Variable	Crude		Adjusted	
	OR	95% CI	OR	95% CI
**Mother age**				
20 ≤ Age ≤ 25	1.00	–	1.00	–
25 < Age ≤ 30	0.90	0.89–0.91	0.87	0.86–0.88
30 < Age ≤ 35	0.96	0.95–0.97	0.90	0.88–0.91
Age > 35	1.11	1.09–1.13	1.01	0.99–1.02
**Monthly income**				
Dependent	1.00	–	1.00	–
< NT$20,000	0.96	0.95–0.97	0.96	0.95–0.98
NT$20,000–$40,000	0.98	0.97–0.99	1.00	0.98–1.01
> NT$40,000	1.13	1.11–1.14	1.13	1.11–1.15
**Individual obstetric history**				
Multifetal pregnancy	9.46	9.15–9.77	9.03	8.73–9.33
Placental abnormality	1.77	1.75–1.79	1.72	1.70–1.74
Diabetes mellitus complicating pregnancy	1.48	1.46–1.50	1.41	1.39–1.42
Vaginal infection	1.06	1.05–1.08	1.00	0.98–1.01
Autoimmune disease	1.35	1.32–1.39	1.29	1.25–1.32
Hypertension complicating pregnancy	3.36	3.29–3.43	3.16	3.09–3.23
Psychological factor(s)	1.28	1.26–1.29	1.25	1.24–1.27
**Periodontal disease**				
No PD	1.00	–	1.00	–
Mild PD	1.08	1.07–1.09	1.05	1.04–1.06
Advanced PD	1.12	1.10–1.14	1.09	1.07–1.11

Regarding the ORs of the 4 age groups for PB and comparing the without, mild, and advanced PD groups after variable adjustment, ORs were significantly higher in the advanced PD group compared with the mild PD group for all age groups and for the no-PD group (OR = 1). The highest ORs for PB in the 2 PD groups were for those aged 31–35 years (OR = 1.09, 95% CI 1.07–1.11 in mild PD group; OR = 1.13, 95% CI 1.09–1.17 in the advanced PD group; Table 3).

**Table 3:** Multivariate analysis of the association between periodontal status and preterm birth according to mother’s age (years).

Variable	20 ≤ Age ≤ 25		25 < Age ≤ 30		30 < Age ≤ 35		Age > 35	
	OR	95% CI	OR	95% CI	OR	95% CI	OR	95% CI
**Monthly income**								
Dependent	1.00	–	1.00	–	1.00	–	1.00	–
< NT$20,000	0.97	0.95–1.00	0.96	0.94–0.98	0.95	0.92–0.97	0.98	0.93–1.03
NT$20,000–$40,000	0.94	0.92–0.96	1.00	0.98–1.03	1.02	0.99–1.05	1.07	1.01–1.12
> NT$40,000	1.05	0.97–1.13	1.14	1.11–1.18	1.13	1.10–1.16	1.15	1.10–1.21
**Individual obstetric history**								
Multifetal pregnancy	7.37	6.77–8.02	9.10	8.61–9.63	9.79	9.26–10.35	8.92	8.10–9.81
Placental abnormality	1.65	1.61–1.69	1.70	1.66–1.73	1.79	1.74–1.83	1.85	1.76–1.94
Diabetes mellitus complicating pregnancy	1.50	1.46–1.55	1.45	1.42–1.48	1.35	1.32–1.38	1.23	1.18–1.28
Vaginal infection	1.00	0.96–1.04	1.00	0.98–1.03	1.01	0.98–1.04	0.96	0.92–1.00
Autoimmune disease	1.20	1.13–1.28	1.26	1.21–1.31	1.33	1.27–1.39	1.38	1.29–1.48
Hypertension complicating pregnancy	2.67	2.55–2.80	3.21	3.09–3.32	3.40	3.26–3.54	3.49	3.29–3.70
Psychological factor(s)	1.33	1.30–1.36	1.25	1.23–1.28	1.21	1.19–1.24	1.19	1.14–1.23
**Periodontal disease**								
No PD	1.00	–	1.00	–	1.00	–	1.00	–
Mild PD	1.00	0.98–1.03	1.06	1.04–1.08	1.09	1.07–1.11	1.03	0.99–1.06
Advanced PD	1.06	1.03–1.10	1.09	1.06–1.12	1.13	1.09–1.17	1.09	1.02–1.15

## Discussion

A population-based cohort study was conducted to investigate associations among age, monthly income, individual obstetric history, PD severity, and the risk of premature delivery. With regard to maternal age, a meta-analysis performed by Pinheiro et al. concluded that women aged over 35 years were generally at a higher risk of adverse obstetrical and perinatal outcomes^36^. That advanced maternal age increases the risk of extreme preterm birth has also been discussed^36–40^. The findings accord with those of the present study, in which the group with a maternal age older than 35 years had the highest preterm birth rate. The maternal age groups of 25–30 years and 30–35 years (OR = 0.87, 95% CI 0.86–0.88; OR = 0.90, 95% CI 0.88–0.91, respectively) were associated with significantly lower risk of PB than the under-25 age group after confounding adjustment (Table 2). Fuchs et al. reported a similar finding for Quebec, Canada. The rate of spontaneous preterm delivery was elevated in women aged 20 to 24 years, and lower prematurity was observed for women aged 30–34 years. The adjusted OR for PB followed a U-shaped distribution from 20 to 24, 25 to 29, 30 to 34, 35 to 39, and 40 or more years^37^.

Regarding PD in relation to PB after age stratification, ORs were found significantly higher in the advanced PD subjects among all age groups (Table 3). We concluded that pregnant women with advanced PD have a higher risk of PB compared to women without PD among all age groups. In the mild PD group, PB risks were also found higher in the groups of 25 < Age ≤ 30 and 30 < Age ≤ 35 but did not show a significant increase in women younger than 25 and older than 35 age groups. In this study, women aged between 25 and 35 years consist of 68% of puerperae, therefore, PD preventions and treatments toward fertile females should be emphasized especially to women with 25–35 years old to reduce the PB rate in Taiwan.

In the present study, women with a higher monthly income had significantly higher risk of PB. However, in studies by Lohana et al. and Morgen et al., SES and preterm delivery did not exhibit a significant association^41,42^. Additionally, controversial results were reported for an investigation conducted by Kramer and Dickute, who concluded that low SES, low educational attainment, and occupation were risk factors for LBW^16,43^. The variables in our database of Taiwan’s NHIRD do not include other SES-related variables such as education, smoking, domicile and occupation. However, studies have found that income inequality might lead to unfavorable health outcomes and increase the risk of preterm birth during pregnancy^44,45^. Some studies have pointed out the relationship between income and health condition correlated with one another in two hypotheses: 1. Income directly affects basic living conditions. 2. Income indirectly affects human health status by influencing their social and living environment^46^. In other words, the less public health services provided by society, the more important personal income is to health.

Taiwan is a country with well-developed medical and health care systems. Multiple policies aid and encourage the public to access personal medical needs. The coverage rate of national health insurance provided by the government reached as high as 99.9%^47^. Additionally, medical accessibility in Taiwan is extremely high compared to other well-developed and developing countries. As for low-income households, the government offers extra medical and premium subsidies. Therefore, the influence of people with low income on medical usage may have less impact in contrast with other countries. The results of this study found that the higher the income, the greater the risk of preterm birth. This might be a result of women with higher salaries and job positions being more engaged to work that develops a higher physical and mental stress of working. We recommend more research and discussion on the relationship between personal income and PB, especially in regions with mature medical insurance systems.

Pregnant women with diabetes or autoimmune or psychological diseases have a higher risk of preterm delivery^48^. Those who have a multifetal pregnancy have the highest risk of preterm delivery, followed by those with hypertension complicating pregnancy after cofounding adjustment^49–51^.

Having a hypertensive disorder during the perinatal period can lead to fetal morbidity, resulting in intrauterine growth restriction and preterm birth. This condition further affects preterm offspring; whether through inherited genetic factors or continued pathophysiology, they have an increased risk of type 2 diabetes, hypertension, and cardiometabolic diseases in adulthood^52,53^. In research by da Silva et al., an increased level of core blood adipokines in preterm infants exposed to maternal hypertension during pregnancy was reported. In addition, a decreased level of ghrelin has been associated with preeclampsia^54,55^.

Periodontitis is a highly prevalent chronic inflammatory disease. Studies have suggested a correlation between multiple systemic diseases such as diabetes, cardiovascular disease, cancer, and dementia and premature delivery^56^; systemic inflammatory responses have been determined to be related to elevated concentrations of circulating inflammatory markers, to form pathogen depositions, neural plaques, and atherosclerosis thrombogenesis, and to induce vascular changes and multiple systemic diseases^22,57,58^. Mediators of systemic inflammation such as cytokines, chemokines, prostaglandins, and C-reactive protein (CRP) have been proved in many studies to be associated with PD^59,60^. Pregnant women with PD and elevated CRP levels being at increased risk for adverse pregnancy outcomes was reported by Ruma et al^61^.

PD affects a large proportion of pregnant woman and occurs in up to 15% of fertile women^41^. The potential causal association between the presence of maternal PD and several adverse pregnancy outcomes is systemic abnormal immunological changes in the fetal/placental unit, which are elevated by PD pathogens and lead to pregnancy complications^28,30,62^. Another hypothesis regarding the mechanism of PD and adverse pregnancy outcomes is that the PD pathogen can colonize the placenta, causing local inflammation and resulting in prematurity and other adverse pregnancy outcomes^63^.

In the present study, primiparas with PD had a higher risk of premature delivery than those without PD. In addition, those with advanced PD had a much higher PB risk compared with those with mild PD. Thus, increased PD severity was related to a higher risk of premature delivery. In the conclusions of Lohana, M. H. et al., PD was a potential risk factor for preterm LBW and, as maternal PD severity increased, the proportion of preterm and LBW deliveries also increased^41^.

According to a study by Albert et al., pregnant women who receive prophylactic dental treatment have a better PD condition and a lower risk of preterm birth than those who do not^64^. In Taiwan, National Health Insurance offers pregnant women an additional free periodontal prophylaxis (in addition to the twice yearly treatment available to all the insured older than 13 years) to prevent PD. The mechanisms underlying the association between PD and premature delivery have not been fully elucidated. PD is a preventable and treatable disease, and pregnant women should improve their oral hygiene to alleviate periodontal tissue inflammation and reduce the risk of PB.

The used of the nationwide population-based database of the NHIRD make the study more credible as it provides sufficient sample size and statistical power to assess the association between PD and PB. However, there are several limitations in the study. The NHIRD is mainly conduct and processed by the Bureau of National Health Insurance (BNHI). In order to prevent miscoding or misclassification by the usage of administrative data, BNHI routinely samples the patient charts from different medical institutes to validate the quality of the database. However, the lack of information of other PB associated factors such as education, occupation, body mass index, and smoking status et al., which cannot be provided from NHIRD, that might reduce accuracy and feasibility of interpreting the analytic outcomes.

## Conclusion

Improving periodontal health before or during pregnancy may prevent or reduce the occurrence of adverse pregnancy outcomes and therefore maternal and perinatal morbidity and mortality. Further studies are required to investigate the mechanism linking PD with adverse pregnancy outcomes. Understanding the impacts of PD on pregnancy can provide empirical evidence for the design of health policy and assist in the development of effective prevention and welfare programs to address adverse pregnancy outcomes.

## Supplementary Information

Supplementary Information.
