# Supplementary material for: Periodontal disease and preterm delivery: a nationwide population-based cohort study of Taiwan
Source: Sci Rep. 2022 Feb 28;12:3297. doi: 10.1038/s41598-022-07425-8 (PMC8885688; doi:10.1038/s41598-022-07425-8)
Supplement: Supplementary file 1 — Supplementary Information. [file 41598_2022_7425_MOESM1_ESM.docx]

**Appendix A**

| Variable | Crude | | Adjusted | |
| --- | --- | --- | --- | --- |
|  | OR | 95% CI | OR | 95% CI |
| Mother age |  |  |  |  |
| 20=<AGE<=25 | 1.00 | - | 1.00 | - |
| 25<AGE<=30 | 0.90 | 0.89-0.91 | 0.87 | 0.86-0.88 |
| 30<AGE<=35 | 0.96 | 0.95-0.97 | 0.89 | 0.88-0.91 |
| AGE>35 | 1.11 | 1.09-1.13 | 1.00 | 0.98-1.02 |
| Socioeconomic status |  |  |  |  |
| Dependent | 1.00 | - | 1.00 | - |
| <NT$20,000 | 0.96 | 0.95-0.97 | 0.96 | 0.95-0.98 |
| NT$20,000-$40,000 | 0.98 | 0.97-0.99 | 1.00 | 0.98-1.01 |
| >NT$40,000 | 1.13 | 1.11-1.14 | 1.13 | 1.11-1.15 |
| Individual obstetric history |  |  |  |  |
| Multifetal pregnancy | 9.46 | 9.15-9.77 | 9.04 | 8.75-9.35 |
| Placental abnormality | 1.77 | 1.75-1.79 | 1.72 | 1.70-1.74 |
| Diabetes mellitus | 1.48 | 1.46-1.50 | 1.41 | 1.39-1.42 |
| Vaginal infection | 1.06 | 1.05-1.08 | 1.00 | 0.98-1.01 |
| Autoimmune disease | 1.35 | 1.32-1.39 | 1.29 | 1.25-1.32 |
| Hypertension complicating pregnancy | 3.36 | 3.29-3.43 | 3.16 | 3.09-3.23 |
| Psychological factor(s) | 1.28 | 1.26-1.29 | 1.26 | 1.24-1.27 |
| Periodontal disease | 1.12 | 1.08-1.15 | 1.06 | 1.05-1.07 |

**Table A1.** Univariate and Multivariate Analysis of Factors Associated with Preterm Birth
